# Associations between metabolic factors and radiographic knee osteoarthritis in early disease - a cross-sectional study of individuals with knee pain

**DOI:** 10.1186/s12891-022-05881-x

**Published:** 2022-10-28

**Authors:** Maria Andersson, E Haglund, K Aili, A Bremander, S Bergman

**Affiliations:** 1 Department of Clinical Sciences, Rheumatology, Lund University, Lund, Sweden; 2 Spenshult research and development centre, Halmstad, Sweden; 3 School of Business, Innovation and Sustainability, Halmstad University, Halmstad, Sweden; 4 School of Health and Welfare, Halmstad University, Halmstad, Sweden; 5 Institute of Environmental Medicine, Karolinska Institutet, Stockholm, Sweden; 6 Department of Regional Health Research, University of Southern Denmark, Odense, Denmark; 7 Danish Hospital for Rheumatic Diseases, University Hospital of Southern Denmark, Sønderborg, Denmark; 8 School of Public Health and Community Medicine / Primary Health Care, Institute of Medicine, Sahlgrenska Academy, University of Gothenburg, Gothenburg, Sweden

**Keywords:** Knee pain, Knee osteoarthritis, Metabolic factors

## Abstract

**Objective:**

Metabolic factors have been shown to be associated to severe radiographic knee osteoarthritis (RKOA). However, more knowledge is needed in early clinical knee osteoarthritis (KOA). The aim was to study associations between metabolic factors and radiographic knee osteoarthritis (OA) in individuals with knee pain. A second aim was to study associations between metabolic factors and RKOA in those with normal BMI and in those overweight/obese, respectively.

**Method:**

This cross-sectional study included 282 individuals with knee pain (without cruciate ligament injury) and aged 30–67 years, and 70% women. Waist circumference, body mass index (BMI), proportion of fat and visceral fat area (VFA) were assessed. RKOA was defined as Ahlbäck grade 1 in at least one knee. Fasting blood samples were taken and triglycerides, cholesterol (total, low density lipoprotein (LDL) and high density lipoprotein (HDL)), C-reactive protein (CRP), glucose, HbA1C were analysed. Metabolic syndrome was defined in accordance with the International Diabetes Federation (IDF). Associations were analysed by logistic regression.

**Results:**

Individuals with RKOA were older, had higher BMI, higher VFA, larger waist circumference and had increased total cholesterol, triglycerides and LDL-cholesterol, but not fasting glucose. There was no difference between the group with RKOA vs. non-radiographic group regarding the presence of metabolic syndrome. In a subgroup analysis of individuals with normal BMI (n = 126), those with RKOA had higher VFA, more central obesity, higher levels of CRP and total cholesterol, compared with individuals without RKOA. In individuals with obesity, age was the only outcome associated to RKOA.

**Conclusion:**

There were clear associations between metabolic factors and RKOA in individuals with knee pain, also in those with normal BMI. In individuals with obesity age was the only variable associated to RKOA.

**Trial registration:**

clinicalTrials.gov Identifier: NCT04928170.

## Introduction

Osteoarthritis (OA) is a group of diseases with various pathophysiological mechanisms, where metabolic, traumatic and age-related phenotypes seem to be significant [1, 2].

The pathophysiological mechanisms in the metabolic phenotype involve factors that are mainly linked to obesity. Obesity affects the weight-bearing joints through increased load and chronic mechanical stress, which induces chondrocytes by mechanoreceptors to synthesize proinflammatory and cartilage-degrading mediators [3, 4]. However, the increased load is probably not the only factor responsible for the association between obesity and OA. The association between hand OA and obesity cannot be explained by mechanical stress [5, 6]. Associations between knee OA, metabolic syndrome (MetS), diabetes type II, increased lipids, and low-grade inflammation have been reported [7–12]. The common factor in these associations might be adipose tissue. Adipose tissue is an endocrine organ and synthesizes adipokines, which are involved in the regulation of glucose, lipids and inflammatory response, etc. [13, 14]. Adipose tissue forms demarcated areas, such as visceral, subcutaneous, skeletal muscular and bone marrow, with dissimilar cellular compositions and possibly different adipokine secretion [13]. Visceral fat is metabolically active and has been reported to be associated to metabolic diseases, such as diabetes type II and dyslipidaemia, and it increases inflammation [15]. Adipokines may have an impact on the development of OA, by exacerbating inflammation, affecting insulin sensitivity and activating cartilage-degrading mechanisms [16]. Studies have also reported associations between glucose levels and OA in the knee, with possible pathophysiological mechanisms, such as oxidative stress in the chondrocytes and advanced glycation end-product (AGE) formation in the cartilage [10]. Several studies report associations between dyslipidaemia and OA, although the mechanisms are not yet fully known [8, 12].

Most studies of metabolic factors in knee OA (KOA) have been investigated in individuals with severe radiographic knee osteoarthritis RKOA. It is suggested that knee pain should be considered as the first sign of what eventually will become RKOA [17]. The hypothesis for this study is that metabolic factors are increased and associated to RKOA. Studies of individuals with knee pain without radiographic changes, representing the early OA disease process are scarce.

The aim of this cross-sectional study was to investigate associations between metabolic factors and RKOA in individuals with knee pain. A second aim was to study associations between metabolic factors and RKOA in those with normal BMI and in those with obesity, respectively.

## Method

### Participants

This cross-sectional study present baseline data from an ongoing five-year longitudinal study, the Halland osteoarthritis cohort (HALLOA), n = 306. The HALLOA cohort profile has been published [18]. The inclusion criteria were knee pain, with no former known RKOA, with a preferred age of 30–65 years. The exclusion criteria were any other rheumatological disorder or previous cruciate ligament injury. Enrolment took place from 2017 to 2019, when seeking care for knee pain in primary health care or by advertisements in local newspapers. A general practitioner examined all participants to confirm the exclusion of rheumatological disorder and previous cruciate ligament injury. Individuals with meniscal lesions or tears were not excluded, as meniscal lesions or tears could be a consequence of the OA process [19]. After examination, nine individuals were excluded due to cruciate ligament injury and two individuals were outside the age range. None of the included individuals had clinically detected synovitis at inclusion. In this study, 282 individuals were included after exclusion of 13 individuals who had not been radiographed or not had blood samples taken at baseline. There were no differences in age or sex between those included in the study (n = 282) and those not included due to missing radiographs or blood samples (n = 13). The study is reported in accordance with the STROBE guidelines.

### Outcome measures

#### Clinical assessments of metabolic factors

Waist circumference was manually assessed with a measuring tape (cm) around the waist, at the height of the navel. Central obesity was classified as waist circumference ≥ 94 cm in men and ≥ 80 cm in women [20]. The height and weight were measured, and BMI was presented as continuous data, but also grouped (underweight < 18.5 kg/m^2^; normal 18.5–24.9 kg/m^2^; overweight 25.0-29.9 kg/m^2^ and obese > 30.0 kg/m2). The two groups BMI < 25 kg/m^2^ and BMI ≥ 25 kg/m^2^, representing those under-/ normal weight as well as overweight/obese was also used. Proportion of fat and visceral fat area (VFA) were assessed by Inbody 770 (Biospace South Korea), which has an intercorrelation coefficient of 0.88-1.00, compared to dual-energy x-ray absorptiometry [21, 22]. Raised VFA levels were classified as VFA ≥ 100cm^2^ [23]. Blood pressure was measured after 5 minutes’ rest (Omron M3, Omron, Sweden). Raised blood pressure was classified as systolic blood pressure ≥ 130 mmHg or diastolic blood pressure ≥ 85 mmHg or treatment of previously diagnosed hypertension [20].

Fasting plasma glucose (mmol/L), triglycerides (TG) (mmol/L), total cholesterol (mmol/L), HDL-cholesterol and LDL-cholesterol (mmol/L), HbA1c (mmol/mol) and C-reactive protein (CRP) > 1.0 mg/L were measured from venous blood, in accordance with the current laboratory standards at Halland County Hospital in Halmstad, Sweden. CRP below 1.0 mg/L was further analysed with a sensitive CRP ELISA method (Abnova). Raised glucose was classified as fasting plasma glucose ≥ 5.6 mmol/L, or previously diagnosed type II diabetes [20]. Raised triglycerides were classified as triglycerides ≥ 1.7 mmol/L or specific treatment for this lipid abnormality [20]. Reduced HDL-cholesterol was classified as HDL-cholesterol < 1.03 mmol/L in males and 1.29 mmol/L in females, or specific treatment for this lipid abnormality [20]. Metabolic syndrome (MetS) was classified in accordance with the International Diabetes Federation (IDF) definition as central obesity plus any two of the following four factors: raised triglycerides, reduced HDL-cholesterol, raised blood pressure, or raised fasting plasma glucose [20].

#### Radiographic assessment

The radiographs were obtained in a skyline view of patellofemoral (PF) joints, and posteroanterior radiographs of both tibiofemoral (TF) joints were obtained in weightbearing position with flexed knees. RKOA was defined in accordance with Ahlbäck [24], i.e. Ahlbäck grade I or more in at least one knee.

### Statistics

To test the differences between groups, the Student’s t-test and the chi-squared test were used where appropriate. Spearman’s rank correlation coefficient was used to assess the relationships between continuous variables. The missing data have not been replaced. All missing data are addressed in the tables. The statistical power was estimated with known prevalence of MetS (14–50%) in previous OA studies, which will give a statistical power of 80% for detecting differences at a 5% significance level in this study. The significance tests were two-tailed and conducted at the 0.05 level of significance.

The associations between outcome factors and RKOA were calculated by univariate logistic regression analyses. Variables associated with RKOA with a significance level of p ≤ 0.25 were separately included in a model adjusting for age and sex [25]. Age was adjusted for sex and sex adjusted for age in this model. Cholesterol and LDL-cholesterol were also controlled for lipid-lowering treatment and HbA1c for treatment for diabetes. Analyses of subgroups were performed by selecting individuals with a normal BMI (BMI < 25) or obese individuals (BMI ≥ 25) and comparing those with and without RKOA.

Statistical analyses were performed using SPSS version 21.0 statistical software (IBM Corp., Armonk, NY, USA).

## Results

A detailed description of the included individuals is shown in Table 1. Besides knee pain, 22% (n = 63) had RKOA at inclusion. About one third fulfilled the criteria for MetS and more than half of the included patients were overweight or obese, and 80% had central obesity. Two thirds had increased blood pressure (whereof 15% were treated for high blood pressure) and more than one third had raised fasting glucose (one individual was treated for diabetes type II). 5% of the included individuals were treated for high lipids.

**Table 1:** Description of the included individuals with no radiographic knee OA (no OA), compared to those with radiographic knee OA (RKOA)

	Missing No OA/OA	All Mean (sd)	No OA Mean (sd)	RKOA* Mean (sd)	p-value
N		282	219	63	
Age, years	0/0	52 (8)	50 (9)	56 (4)	< 0.001
Sex, women, %	0/0	70	70	68	0.81
BMI	0/0	26.4 (4.7)	26.5 (4.8)	27.8 (4.2)	0.011
BMI < 18.5 kg/m^2^, %	0/0	1	1	0	
BMI 18.5–24.9 kg/m^2^, %	0/0	44	47	33	0.147
BMI 25.0-29.9 kg/m^2^, %	0/0	35	33	45	
BMI > 30.0 kg/m^2^, %	0/0	20	19	22	
Underweight and normal weight, %	0/0	45	48	32	0.019
Overweight and obese, %		55	52	68	
Visceral fat area (VFA)	4/1	114 (53)	109 (53)	128 (51)	0.014
Raised VFA (≥ 100), %	4/1	51	46	68	0.003
Proportion of fat, %	4/1	31 (9)	30 (9)	33 (9)	0.042
Waist circumferance, cm	3/1	95 (13)	94 (13)	99 (12)	0.011
Central obesity, %	3/1	81	76	94	0.001
Raised blood pressure**, %	1/0	63	64	57	0.31
CRP, mg/L	0/0	2.0 (2.6)	1.9 (2.6)	2.3 (2.4)	0.33
Total cholesterol, mmol/L	0/0	5.3 (1.1)	5.2 (1.1)	5.5 (1.0)	0.053
Triglycerides, mmol/L	0/0	1.1 (0.6)	1.0 (0.6)	1.2 (0.7)	0.11
Raised triglycerides**, %	0/0	12	10	19	0.041
LDL-cholesterol, mmol/L	0/0	3.5 (1.0)	3.4 (1.0)	3.7 (1.1)	0.046
HDL-cholesterol, mmol/L	0/0	1.7 (0.4)	1.7 (0.4)	1.7 (0.5)	0.75
Reduced HDL-cholesterol**	0/0	13	12	14	0.69
Glucose, mmol/L	2/0	5.4 (0.8)	5.4 (0.9)	5.4 (0.5)	0.97
Raised glucose**, %	2/0	34	35	33	0.84
HBA1c, mmol/mol	9/1	37 (5)	37 (5)	37 (4)	0.77
MetS**, %	4/1	27	26	34	0.20

RKOA, radiographic knee osteoarthritis; BMI, body mass index; CRP, C-reactive protein; LDL, LDL, low density lipoprotein; HDL, high density lipoprotein; HbA1C, glycosylated hemoglobin; MetS, metabolic syndrome; KOOS, knee injury and osteoarthritis outcome score; ADL, Function in daily living; Sport/recreation, Function in sport and recreation; QoL, knee-related quality of life

*Ahlbäck grade I or more in at least one knee

**according to International Diabetes Federation (IDF)

### Radiographic knee osteoarthritis

The group with RKOA (n = 63) were on average older, had higher VFA, central obesity, higher total cholesterol and LDL-cholesterol and were more likely to have raised TG, than the group without RKOA. No differences were seen for CRP levels, glucose levels and there was no difference in proportion of MetS between the groups, Table 1.

In a univariate logistic regression, age, BMI, VFA, waist circumference, central obesity, proportion of fat, raised TG and LDL-cholesterol were associated with RKOA, Table 2. In the multivariate logistic regression model, raised VFA and central obesity were associated with having RKOA, Table 2.

**Table 2:** Associations with radiographic knee osteoarthritis(RKOA, Ahlbäck grade I or more in at least one knee) analysed with two logistic regression models. One univariate and one adjusting for age and sex

	Univariate logistic regression			Adjusting for age and sex		
	OR	95% CI	p-value	OR	95% CI	p-value
Age, years	1.13	1.07,1.19	< 0.001	1.13	1.07, 1.19	< 0.001
Sex, women	0.93	0.51,1.70	0.81			
BMI	1.08	1.02,1.14	0.013	1.06	1.00,1.13	0.06
BMI ≥ 25, kg/m^2^	2.02	1.12,3.65	0.020	1.87	0.99,3.52	0.05
Visceral fat area (VFA), cm^2^	1.01	1.00,1.01	0.016	1.01	1.00,1.01	0.08
Raised VFA (≥ 100), cm^2^	2.46	1.36,4.47	0.003	2.43	1.29,4.55	0.006
Proportion of fat, %	1.03	1.00,1.07	0.044	1.03	0.99,1.07	0.11
Waist circumferance, cm	1.03	1.00,1.05	0.012	1.02	1.00,1.05	0.06
Central obesity*	6.08	1.83,20.22	0.003	5.19	1.52,17.72	0.009**
Raised blood pressure*	0.74	0.42,1.31	0.31			
CRP, mg/L	1.05	0.95,1.16	0.34			
Total cholesterol, mmol/L	1.29	1.00,1.66	0.055	1.08	0.81,1.43	0.61***
Triglycerides, mmol/L	1.40	0.92,2.14	0.12	1.19	0.75, 1.90	0.46***
Raised triglycerides*	2.21	1.02,4.78	0.045	1.92	0.84,4.36	0.12
LDL-cholesterol, mmol/L	1.31	1.00,1.71	0.048	1.12	0.84,1.50	0.45***
Reduced HDL-cholesterol*	1.18	0.52,2.66	0.69			
Glucose, mmol/L	0.99	0.70,1.40	0.97			
Raised glucose*	0.94	0.52,1.70	0.84			
HBA1c, mmol/mol	1.01	0.95,1.07	0.77			
MetS*	1.49	0.81,2.74	0.20			

BMI, body mass index; CRP, C-reactive protein; LDL, low density lipoprotein; HDL, high density lipoprotein; HbA1C, glycosylated hemoglobin; MetS, metabolic syndrome

*according to International Diabetes Federation (IDF) **controlled only for age *** Triglycerides, cholesterol and LDL-cholesterol were also controlled for lipid-lowering treatment

### Subgroup analysis

Of those with normal BMI (< 25 kg/m^2^, n = 126), 16% had RKOA, 20% had raised fasting glucose, and 58% had central obesity. Those who had RKOA (n = 20) were older, had a higher rate of raised VFA, central obesity and total cholesterol, compared with those who did not fulfil the criteria for RKOA (n = 106), Table 3.

**Table 3:** Description of the subgroup with normal BMI (n = 126), with and without radiographic knee osteoarthritis (RKOA)

	Missing No OA/OA	No OA Mean (sd)	RKOA* Mean (sd)	p-value
N		106	20	
Age, years	0/0	50 (9)	56 (4)	< 0.001
Sex, women, %	0/0	78	80	0.86
Visceral fat area (VFA), cm^2^	2/0	72 (25)	89 (30)	0.008
Raised VFA, (≥ 100 cm^2^), %	2/0	9	40	< 0.001
Proportion of fat, %	2/0	25 (6)	27 (8)	0.11
Waist circumferance, cm	1/1	84 (8)	87 (7)	0.12
Central obesity, %	1/1	53	84	0.012
Raised blood pressure**, %	0/0	75	55	0.08
CRP, mg/L	0/0	1.1 (1.0)	2.1 (2.7)	0.005
Total cholesterol, mmol/L	0/0	4.9 (1.0)	5.6 (1.0)	0.012
Triglycerides, mmol/L	0/0	0.9 (0.4)	0.9 (0.5)	0.82
Raised triglycerides**, %	0/0	3	5	0.62
LDL-cholesterol, mmol/L	0/0	3.1 (0.8)	3.6 (1.2)	0.06
HDL-cholesterol, mmol/L	0/0	1.8 (0.4)	2.0 (0.5)	0.035
Reduced HDL**	0/0	6	5	0.90
Glucose, mmol/L	2/0	5.2 (0.5)	5.2 (0.4)	0.72
Raised glucose**, %	2/0	20	20	0.97
HBA1c, mmol/mol	3/0	36 (3)	37 (3)	0.11
MetS**, %	1/1	12	16	0.68

RKOA, radiographic knee osteoarthritis; BMI, body mass index; CRP, C-reactive protein; LDL, low density lipoprotein; HDL, high density lipoprotein; HbA1C, glycosylated hemoglobin; MetS, metabolic syndrome

*Ahlbäck grade I or more in at least one knee **according to International Diabetes Federation (IDF)

In individuals with normal BMI, there were associations between RKOA and older age, higher VFA, central obesity, higher CRP, total cholesterol and LDL-cholesterol, Table 4. In the multivariate logistic regression, raised VFA was associated to RKOA.

**Table 4:** Associations with radiographic knee osteoarthritis(RKOA, Ahlbäck grade I or more in at least one knee) in the subgroup with normal BMI (n = 126) analysed with two logistic regression models. One univariate and one adjusting for age and sex

	Univariate logistic regression			Adjusting for age and sex		
	OR	95% CI	p-value	OR	95% CI	p-value
Age, years	1.12	1.04,1.22	0.006	1.13	1.04, 1.22	0.006
Sex, women	1.11	0.34,3.64	0.86			
Visceral fat area (VFA), cm^2^	1.02	1.00,1.04	0.017	1.02	1.01,1.04	0.014
Raised VFA (≥ 100), cm2	7.04	2.28,21.69	0.001	9.58	2.55,35.94	0.001
Proportion of fat, %	1.06	0.99,1.15	0.11	1.09	0.98, 1.22	0.11
Waist circumferance, cm	1.04	0.99,1.10	0.13	1.06	0.99, 1.13	0.08
Central obesity*	3.50	1.10,11.17	0.034	2.93	0.89,9.69	0.08**
Raised blood pressure*	0.42	0.16,1.12	0.08	0.51	0.18,1.44	0.20
CRP, mg/L	1.39	1.06,1.82	0.017	1.33	0.98,1.82	0.07
Total cholesterol, mmol/L	1.79	1.12,2.86	0.016	1.58	0.95,2.63	0.08
Triglycerides, mmol/L	1.14	0.39,3.34	0.82			
Raised triglycerides*	1.79	0.18,18.13	0.62			
LDL-Cholesterol, mmol/L	1.61	0.98,2.64	0.06	1.45	0.85,2.47	0.18
Reduced HDL-Cholesterol mmol/L*	0.87	0.10,7.63	0.90			
Glucose, mmol/L	0.83	0.30,2.60	0.72			
Raised glucose*	0.98	0.30,3.23	0.97			
HbA1c, mmol/mol	1.13	0.97,1.31	0.11	1.07	0.90, 1.26	0.44
MetS*	1.25	0.32,4.86	0.75			

CRP, C-reactive protein; LDL, low density lipoprotein; HDL, high density lipoprotein; HbA1C, glycosylated hemoglobin; MetS, metabolic syndrome

*according to International Diabetes Federation (IDF) **controlled only for age ***. Cholesterol and LDL-cholesterol was also controlled for lipid-lowering treatment and HbA1c for treatment for diabetes

In another subgroup analysis of those with overweight/obesity, BMI above 25 kg/m^2^, older age was the only parameter that was associated with RKOA (Tables 5 and 6).

**Table 5:** Description of the overweight/obese (BMI ≥ 25, n = 156) subgroup, with and without radiographic knee osteoarthritis (RKOA)

	Missing No OA/OA	No OA Mean (sd)	RKOA* Mean(sd)	p-value
N		113	43	
Age, years	0/0	51 (8)	56 (4)	< 0.001
Sex, women, %	0/0	62	63	0.92
Visceral fat area (VFA), cm^2^	2/1	144 (48)	147 (49)	0.79
Raised VFA, %	2/1	81	81	0.99
Proportion of fat, %	2/1	35 (9)	35 (8)	0.84
Waist circumferance, cm	2/0	104 (10)	104 (10)	0.79
Central obesity, %	2/0	98	100	0.38
Raised blood pressure**, %	1/0	54	58	0.68
CRP, mg/L	0/0	2.7 (3.3)	2.4 (2.4)	0.56
Total cholesterol, mmol/L	0/0	5.4 (1.1)	5.5 (1.0)	0.89
Triglycerides, mmol/L	0/0	1.2 (0.7)	1.3 (0.7)	0.34
Raised triglycerides**, %	0/0	16	26	0.17
LDL, mmol/L	0/0	3.7 (1.1)	3.8 (1.0)	0.62
HDL, mmol/L	0/0	1.6 (0.4)	1.5 (0.4)	0.24
Reduced HDL**	0/0	19	19	0.99
Glucose, mmol/L	0/0	5.7 (1.1)	5.6 (0.6)	0.60
Raised glucose**, %	0/0	48	39	0.36
HBA1c, mmol/mol	6/1	38 (6)	37 (4)	0.42
MetS**, %	3/0	38	42	0.68

RKOA, radiographic knee osteoarthritis; BMI, body mass index; CRP, C-reactive protein; LDL, low density lipoprotein; HDL, high density lipoprotein; HbA1C, glycosylated hemoglobin; MetS, metabolic syndrome

*Ahlbäck grade I or more in at least one knee **according to International Diabetes Federation (IDF)

**Table 6:** Associations with radiographic knee osteoarthritis (RKOA), according to Ahlbäck grade I, in the subgroup with overweight/obesity (BMI ≥ 25, n = 156) according to body mass index (BMI). Two models were used, one univariate and one adjusting for age and sex

	Univariate logistic regression			Adjusting for age and sex		
	OR	95% CI	p-value	OR	95% CI	p-value
Age, years	1.14	1.06,1.22	< 0.001	1.14	1.06, 1.22	< 0.001
Sex, women	1.04	0.50,2.14	0.92			
Visceral fat area (VFA), cm^2^	1.00	0.99,1.01	0.79			
Raised VFA (≥ 100), cm2	0.99	0.40,2.45	0.99			
Proportion of fat, %	1.00	0.96,1.05	0.84			
Waist circumferance, cm	1.01	0.97,1.04	0.78			
Central obesity*	**--**					
Raised blood pressure*	1.16	0.57,2.36	0.68			
CRP, mg/L	0.96	0.84,1.10	0.56			
Total cholesterol, mmol/L	1.02	0.74,1.41	0.88			
Triglycerides, mmol/L	1.26	0.78,2.06	0.34			
Raised triglycerides*	1.81	0.78,4.25	0.17	1.55	0.62, 3.88	0.35**
LDL-Cholesterol, mmol/L	1.09	0.78,1.52	0.62			
Reduced HDL-Cholesterol mmol/L*	1.00	0.41,2.47	0.99			
Glucose, mmol/L	0.89	0.58,1.37	0.61			
Raised glucose*	0.71	0.35,1.46	0.36			
HbA1c, mmol/mol	0.97	0.89,1.05	0.42			
MetS*	1.17	0.57,2.39	0.68			

BMI, body mass index; CRP, C-reactive protein; LDL, low density lipoprotein; HDL, high density lipoprotein; HbA1C, glycosylated hemoglobin; MetS, metabolic syndrome

*according to International Diabetes Federation (IDF) **controlled for age

## Discussion

This is a study of individuals with knee pain, were also those without radiographic changes, supposedly representing a group with increased probability for being in an early OA disease process [17]. Several metabolic factors were more common in participants with RKOA, also found in individuals with a normal BMI.

There was a high prevalence of individuals with central obesity, increased VFA and overweight/obese (BMI > 25 kg/m^2^) in this study group, as was to be expected. Obesity is a risk factor for knee OA [26, 27], and it is also well known that individuals with knee pain have a high risk of developing RKOA over time, regardless of whether they are overweight or not [17]. A previous study suggests that MetS and its components are causing the knee pain. [28]. However, in this study, only central obesity and VFA were associated with RKOA when adjusting for age and sex, suggesting that visceral fat is an important factor. The impact of visceral fat needs to be studied in a longitudinal setting to be able to study causal relationship. Also, individuals with a normal BMI and RKOA had a higher visceral fat, higher cholesterol levels and higher CRP, compared to those with normal BMI and no radiographic OA. These findings are supported by a newly published study in mice which reported a direct association between adipose tissue and cartilage damage, independent of body weight [29]. For individuals with normal BMI mechanical stress is probably not the main cause of their RKOA, as suggested for obese individuals [30, 31]. Also, in individuals with normal BMI, metabolic factors and visceral fat seem to be more important [32]. Visceral fat measure, such as VFA and central obesity, seems to be more important factors than BMI, when evaluating individuals with knee pain, and risk of having RKOA. BMI does not reflect the body fat mass or amount of visceral fat [33].

In this study, those with RKOA were older, and age is a well-known risk factor for RKOA [34]. When getting older the distribution of visceral fat changes, but if this is causing the increased risk or whether it is the metabolic load over a longer period of time, needs to be elucidated. There was no association between RKOA and sex in this study. A Meta-analysis has found men to have a lower risk of knee OA, especially for those aged 55 and above [35]. However, the criteria for having knee OA differed between the studies included in the meta-analysis, from clinical to radiological with and without symptoms, which could have affected the results [35]. Another study reported women experiencing more pain regardless of radiographic severity [36], and could fulfil symptom criteria due to more knee pain.

About one fifth of the individuals without RKOA fulfilled the IDF criteria for MetS which is a percentage that is comparable to the general population (22–28%) [37]. One third of those with RKOA fulfilled the criteria for MetS, which is fewer than reported by other studies (43–59%) [7, 38]. An explanation for this disparity between the data in the present study and other studies could be the use of different criteria for MetS. The World Health Organization (WHO) and European Group for the study of Insulin Resistance (EGIR) definitions include insulin resistance in the criteria, whereas the IDF definition has waist circumference as a mandatory component, which could explain the disparity.

About two thirds of the participants had raised blood pressure, with no differences between those with and without RKOA, which is in line with previous studies of individuals with radiological knee OA [7, 39]. In other studies, hypertension or cardiovascular diseases are reported to be linked to OA as elements of the pathological mechanisms in OA [40–42]. One possible explanation between the divergent results could be that our participants were in early disease course and only 22% had RKOA.

One third of the individuals in the present study had raised fasting glucose levels, according to the IDF criteria; however, only one of the included individuals had treatment for diabetes (type II). The cut-off for raised fasting glucose in Sweden is 6.0 mol/L and the cut-off for raised fasting glucose in the IDF criteria is 5.6 mmol/L, which could explain why most of the participants were untreated for their raised fasting glucose. The mean value of fasting glucose in the present study is in the upper part of the normal range in Sweden (4.0–6.0 mmol/L). In the present study, fasting glucose levels were not associated with RKOA, as reported in other studies [43, 44]. One possible explanation could be that our participants were in early disease course, and few had RKOA.

In the group with normal BMI, those with RKOA had higher level of CRP. CRP was also associated with RKOA in the univariate model and showed a borderline significance in the multivariate logistic regression. The increased inflammation measured by serum CRP is probably caused both by synovial inflammation and inflammation due to obesity [45–48]. The synovial inflammation in OA is already seen in early stages of the disease and increases in later stages [49].

Lipids were increased in those with RKOA in the present study and total cholesterol, LDL-cholesterol and triglyceride levels were associated with RKOA. Dyslipidaemia has been reported in previous studies and has been suggested to be involved in the pathogenesis of OA [8, 12, 50].

In individuals with overweight/obesity, 28% had RKOA. There were no differences in metabolic factors between the groups with and without RKOA. This finding could be explained by the fact that dyslipidaemia and diabetes type II are linked to obesity and both groups were overweight or obese [51, 52]. Because of obesity, the group without radiological knee OA are also at high risk for developing RKOA.

One fifth of participants in the present study had RKOA, and most had not had a radiographic examination of their knees before study start. The rareness of the studied cohort, still early in the disease course and with a high risk of developing knee OA, will be accentuated in longitudinal follow-up studies.

A limitation in this study was that magnetic resonance imaging (MRI) was not assessed to evaluate the knees, which could have affected the result. The radiographs were scored according to Ahlbäck and not to Kellgren and Lawrence, which is more common. In this study, the aim was to get a cut off for RKOA and Ahlbäck grade I is comparable to Kellgren & Lawrence grade II [53]. The use of Ahlbäck grade I or more as cut off for RKOA could have affected the result. One of the exclusion criteria was previous cruciate ligament injury diagnosed clinically or by MRI at time for injury; no other knee injuries were included in this exclusion criterion, which also could have influenced the results. Individuals with meniscal lesions or tears were not excluded from the study, which could have influenced the results. Inbody 770 was used instead of DXA, which is the gold standard. This could have influenced the results, however, Inbody 770 has a high correlation to DXA [21] and is a cheaper and easier to use. In the subgroup analyses some of the groups have a small sample size (n = 20 and n = 43), which could have had an impact on the result. However, there were differences in the subgroup analysis and associations were found. All associations found in the subgroup analysis are also found in the whole group. Recruitment in which participants themselves announces interest for enrolment can be a limitation. To minimize this limitation, all participants were examined by a physician at the start of the study. It could although to some extent reduce the generalisability of the results. The number of comparisons in the study increases the risk of rejecting a true null hypothesis, and caution is necessary when interpreting statistical significance. However, this will not alter the interpretation of the main findings. This study is a part of a five-year longitudinal study with yearly interventions, which affords the opportunity to study the disease progress thoroughly.

## Conclusion

Metabolic factors were associated with RKOA, in this study, also in those with normal BMI. For individuals with normal BMI, increased load and mechanical stress is probably not the main cause of their RKOA; instead, metabolic factors could be of importance. The associations between metabolic risk factors and development of RKOA in individuals with knee pain, especially in those with normal BMI, needs to be assessed in longitudinal studies, with a focus on pathophysiological mechanisms.

## Data Availability

The datasets used and/or analyzed during the current study are available from the corresponding author on reasonable request.

## Abbreviations

ADL: Function in daily living
AGE: advanced glycation end-product
BMI: body mass index
CRP: C-reactive protein
ELISA: enzyme-linked immunosorbent assay
IDF: International Diabetes Federation
HALLOA: the Halland osteoarthritis cohort
HbA1C: glycosylated hemoglobin
HDL: high density lipoprotein
KOOS: knee injury and osteoarthritis outcome score
LDL: low density lipoprotein
MetS: metabolic syndrome
MRI: magnetic resonance imaging
OA: osteoarthritis
PF: patellofemoral
RKOA: radiographic knee osteoarthritis
TF: tibiofemoral
VFA: visceral fat area
